# Amino acid consumption in naïve and recombinant CHO cell cultures: producers of a monoclonal antibody

**DOI:** 10.1007/s10616-014-9720-5

**Published:** 2014-05-06

**Authors:** L. M. Carrillo-Cocom, T. Genel-Rey, D. Araíz-Hernández, F. López-Pacheco, J. López-Meza, M. R. Rocha-Pizaña, A. Ramírez-Medrano, M. M. Alvarez

**Affiliations:** Centro de Biotecnología-FEMSA, Tecnológico de Monterrey at Monterrey, Av. Eugenio Garza Sada 2501 sur. Col. Tecnológico. Edificio CB, 5to Piso, 64849 Monterrey, NL Mexico

**Keywords:** CHO cells, Amino acid consumption, Monoclonal antibodies, Biopharmaceutical, Feed strategies, Mammalian cell culture

## Abstract

Most commercial media for mammalian cell culture are designed to satisfy the amino acid requirements for cell growth, but not necessarily those for recombinant protein production. In this study, we analyze the amino acid consumption pattern in naïve and recombinant Chinese hamster ovary (CHO) cell cultures. The recombinant model we chose was a CHO-S cell line engineered to produce a monoclonal antibody. We report the cell concentration, product concentration, and amino acid concentration profiles in naïve and recombinant cell cultures growing in CD OptiCHO™ medium with or without amino acid supplementation with a commercial supplement (CHO CD EfficientFeed™ B). We quantify and discuss the amino acid demands due to cell growth and recombinant protein production during long term fed batch cultivation protocols. We confirmed that a group of five amino acids, constituting the highest mass fraction of the product, shows the highest depletion rates and could become limiting for product expression. In our experiments, alanine, a non-important mass constituent of the product, is in high demand during recombinant protein production. Evaluation of specific amino acid demands could be of great help in the design of feeding/supplementation strategies for recombinant mammalian cell cultures.

## Introduction

Mammalian cell culture plays a key role in the biopharmaceutical industry. For example, Chinese hamster ovary (CHO) cells and other mammalian cell lines are routinely used for the production of recombinant proteins of therapeutic interest, such as monoclonal antibodies, erythropoietin, etc. Among the 58 biopharmaceuticals approved from 2006 to 2010, 32 are produced in mammalian cells (Marichal-Gallardo and Alvarez 2012; Walsh 2010) and nearly 70 % of all recombinant therapeutic proteins are produced in Recombinant Chinese hamster ovary (rCHO) cells (Jayapal et al. 2007).

Chinese hamster ovary cells have been the most widely used mammalian host for large-scale commercial production of recombinant proteins when a safe host and efficient post-translational modifications are required; therefore, these cells are very important to the modern biopharmaceutical industry (Durocher and Butler 2009). Recent advances in cell culture technology for rCHO cells have resulted in significant improvements in terms of protein titer to meet the market’s continuously growing needs. This improvement in protein titer is mainly due to the development of stable high producers through vector design and host cell engineering as well as through optimization of the culture processes (Kim et al. 2012). Optimization of the culture medium is an obvious central activity in the design of biopharmaceutical processes. However, even after decades of industrial practice, culture medium design and optimization continue to have opportunities for improvement. No general protocols exist for cell culture medium optimization and several approaches are used in the industry. In particular, reports on optimization of culture media or feeding strategies for CHO cell cultures are limited in number. These strategies are commonly based on statistical design (Castro et al. 1992; Lee et al. 1999; Parampalli et al. 2007; Zhang et al. 2013), and are primarily focused on optimizing the carbon and energy metabolism (Lu et al. 2005; Tsao et al. 2005; Wilkens et al. 2011), on the addition of complex supplement free from animal components (Tang et al. 2008; Mosser et al. 2013), and on medium and feeding strategy development (Li et al. 2005; Zhang et al. 2004, 2013, Rouiller et al. 2013). Recently, examples of experiments where nutrients (mainly carbon sources) have been supplemented according to depletion rates measured online (or calculated) have become available (Sellick et al. 2011; Lu et al. 2013). Although amino acid supplementation is recognized as one of the crucial parameters in cell culture medium design and optimization, only a few reports focus on this particular theme (Jordan et al. 2013; González et al. 2011; Quek et al. 2010; Xing et al. 2011; Tang et al. 2008; Zhang 2009; Altamirano et al. 2006). Amino acids are among the most important nutrients for promoting cell growth and increases in productivity, since they constitute a nitrogen source and are the building blocks of proteins (both native and recombinant), as well as intermediaries of several metabolic pathways. Through anabolism, they form proteins, polypeptides, and other nitrogen related compounds, while energy to support growth and survival comes from their catabolism.

Most commercial media for mammalian cell culture are designed to satisfy the amino acid requirements for cell growth, but not necessarily those for recombinant protein production. Therefore, amino acid supplementation is needed to maximize recombinant protein production. Evidently, the amino acid demand for protein expressions is product specific, since it depends on the molecular structure of each protein. In addition, some amino acids are important for the appropriate biochemical functioning of recombinant mammalian cells, even though they may not necessarily be main constituents of the recombinant product. Several reports are available on the effects of particular amino acids on different aspects of CHO cell metabolism. For example, the addition of the amino acids threonine, proline, and glycine improved CHO cell growth by impacting metabolic parameters such as glucose consumption, lactate production, glutamine utilization, and final ammonium levels, as well as by enhancing the production levels of recombinant tissue plasminogen activator (t-PA) (Chen and Harcum 2005). Experiments by Altamirano et al. (2004) showed lowered ammonia levels when glutamate was used instead of glutamine. Supplementation with proline, serine, and asparagine in the culture medium enhanced cell growth, while asparagine supplementation also improved the production and biological quality of t-PA (Altamirano et al. 2006). Hybridoma and CHO cells grown in the presence of elevated pCO_2_ are also protected by asparagine and glycine (DeZengotita et al. 2002). The influence of amino acid resources on cell proliferation and monoclonal antibody secretion has been studied previously—for example, in hybridoma cells—by determining the pattern of amino acid consumption in two different cell lines (Duval et al. 1991). The amino acid supply was then confirmed as one of the factors limiting cell growth and productivity in batch processes. In the case of a specific CHO-DG44 cell line, González et al. (2011) used a Plackett-Burman statistical design to evaluate the effect of selected amino acids on cell growth. The authors found that leucine and arginine had the highest negative and positive effects, respectively, on cell viability, while leucine and threonine addition resulted with the highest negative effects on growth rate, and valine and arginine demonstrated the highest positive impact on final mAb concentration.

The design of amino acid supplementation strategies might be significantly simplified by determining the actual amino acid demands of a cell culture due to cell growth and recombinant protein production. In this study, we analyzed the amino acid consumption for a naïve and a recombinant CHO-S cell line producer of a anti TNFα monoclonal antibody. We discuss the amino acid demands due to cell growth and recombinant protein production during long term fed batch cultivation protocols. We followed amino acid concentrations using an AccQ-Tag pre-column derivatization technique followed by high performance liquid chromatography coupled to fluorescence detection. This method has been applied to characterize free and total amino acid profiles from culture media and fermentation broths (Aliaga 2010; Fiechter and Mayer 2011; González et al. 2011; Zagari et al. 2013). This technique has also been applied to highly sensitive amino acid analysis of sub-picomolar amount of proteins while maintaining quantitative performance (Masuda and Dohmae 2011).

## Materials and methods

The main objective of this work was to determine the amino acid demands for a specific CHO cell line, through an actual cell culture process, and to propose this strategy as a way to assist in the rational design of culture media for biopharmaceutical applications.

### Experimental design and culture media

**Fig. 1:**
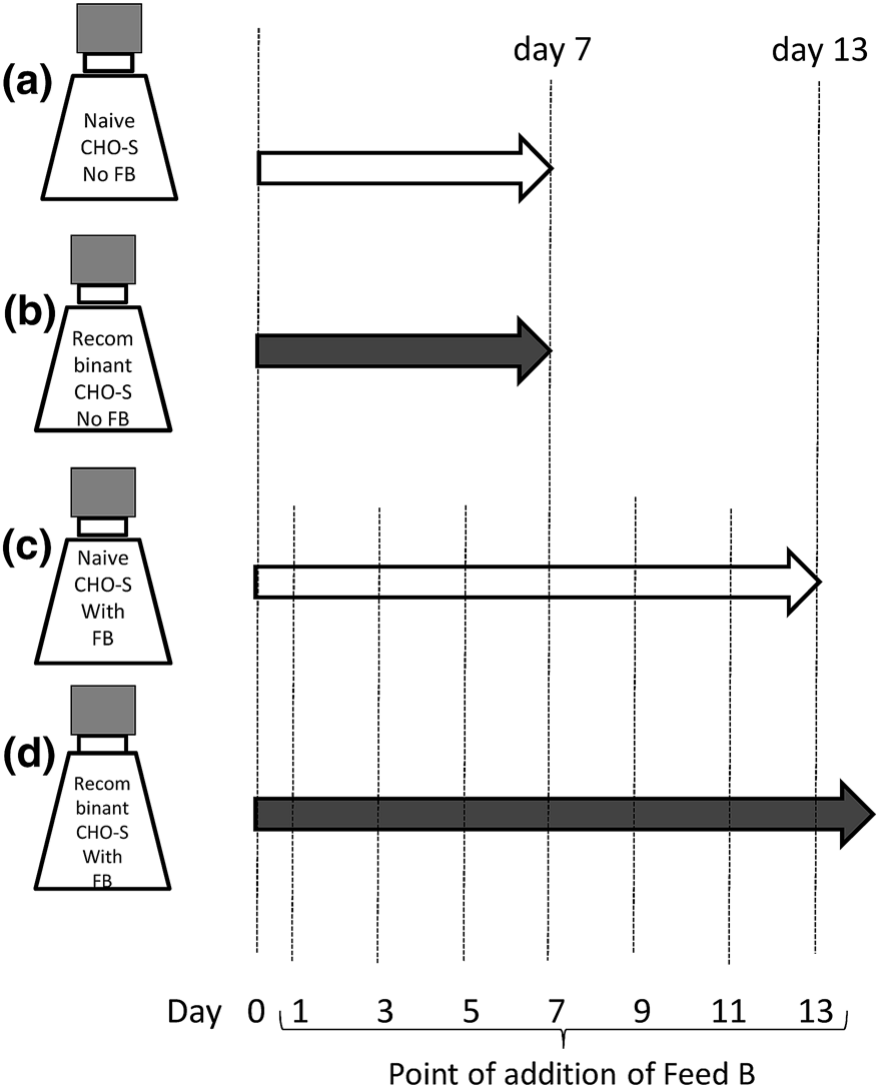
Scheme of the experimental design used. Naïve and recombinant (producing a biosimilar of a commercial anti-TNFα mAb) CHO cells were cultured in batch mode (without amino acid supplementation) and in fed-batch mode (with amino acid supplementation). Four different treatments were explored: **a** naïve CHO cells cultured in CD OptiCHO™ medium without supplementation; **b** mAb producer cells cultured in CD OptiCHO™ medium without supplementation; **c** naïve CHO cells cultured in CD OptiCHO™ medium and supplemented with CHO CD EfficientFeed™ B (FB); and **d** mAb producer cells cultured in CD OptiCHO™ medium and supplemented with CHO CD EfficientFeed™ B (FB)

Naïve and recombinant (producing a monoclonal antibody) CHO cells were cultured in 125 mL culture flasks in batch mode (without any supplementation), and fed-batch mode (supplemented with CHO CD EfficientFeed™ B (catalog number A10240-01 from Gibco™, Life Technologies, Carlsbad, CA, USA) on days 1, 3, 5, 7, 9, 11 and 13). This gave a total of four experimental treatments (see Fig. 1): (a) naïve cells cultured in batch mode without supplementation; (b) naïve cells cultured in fed-batch mode with supplementation; (c) recombinant cells cultured in batch mode without supplementation; and (d) recombinant cells cultured in fed-batch mode with supplementation. At least three repetitions of each treatment were conducted. All experiments used CD OptiCHO™ medium (cat. 12681-029, Invitrogen, Carlsbad, CA, USA) and l-glutamine (cat. 25030-164, Invitrogen) was added up to a final concentration of 8 mM.

### Cell line and cell culture conditions

The naïve CHO cells were adapted to grow in suspension (original cell line: CHO-S, cat. R800-07, Invitrogen). The recombinant clone (see also González et al. 2011), a producer of an anti-TNFa biosimilar (biosimilar of Infliximab™), was derived in-house from the naïve CHO cell line. The genetic construction used included an UCOE™ (from Millipore, Billerica, MA, USA) sequence and an IRES sequence.

All culture experiments were initiated by seeding 30 mL of CD OptiCHO™ medium with 2 × 10^5^ cell/mL. In experiments with supplementation, a volume of 3 mL of CHO CD EfficientFeed™ B was added to each culture on days 1, 3, 5, 7, 9, 11 and 13. All cultures were incubated at 33 °C during the first five days. Subsequently, the temperature was lowered to 31 °C at day 5. Sampling was made on a daily basis by removing 1 mL aliquots, which were stored at −20 °C prior to analysis. On days 1, 3, 5, 7, 9, 11 and 13, sampling was done before supplementation. Cellular density, viability, pH, and osmolality were determined (data not shown). Experiments ceased when cell viability dropped below 50 %; this occurred between day 7 and 8 in experiments without supplementation, and at day 13 or 15 in experiments with supplementation. Cell density and cell viability were determined by cell counting using a guava^®^easyCyte™ 8HT flow cytometer (Millipore).

### mAb quantitation by indirect ELISA

The concentration of active mAb (i.e., capable of recognizing TNFα) was determined by an ELISA procedure, as previously described by González et al. (2011). Briefly, samples form mAb producer CHO cell cultures were centrifuged at 5,000 rpm for 5 min to remove cells and cellular debris. Supernatants were diluted using PBS 1X to a 1:1 ratio. In 96 well plates (Maxisorp; NUNC, New York, NY, USA), each of the following solutions were added sequentially: 100 μL/well of a 5 μg/mL TNFα (BioSource™; Invitrogen), 300 μL/well of SuperBlock^®^T20 (Thermo Scientific; Pierce, Rockford, IL, USA), 100 μL/well of samples or standards [standards were prepared by serial dilution from commercially available Infliximab (Remicade^®^; Schering-Plough, Innishannon, Ireland)], 100 μL/well of anti-human IgG-HRP conjugate at a 1:30,000 dilution (Thermo Scientific; Pierce), and 100 μL/well of 1-step Ultra TMB-ELISA (Thermo Scientific; Pierce). In between the addition of the different solutions, individual wells were washed three times with 300 μL/well of PBS-0.05 % Tween-20 solution (10 mM phosphate, 0.15 M NaCl, pH 7.2 ± 0.2). The TNFα solution was incubated at 4 °C overnight. The rest of the incubation steps were done at room temperature for 1 h. The enzymatic reaction was stopped by adding 50 μL/well of 1 M H_2_SO_4_. Plates were read at 450 nm.

### Amino acid analysis by pre-column AQC derivatization

Pre-column amino acid derivatization with 6-aminoquinolyl-N-hydroxysuccinimidyl carbamate (AQC) was performed for commercial culture medium, commercial supplement, and CHO cell culture samples (from both naïve and recombinant experiments) taken at different time points following the Waters AccQ.Tag™ methodology (Waters AccQFluor Reagent Kit from Waters™ (cat num. WAT052875, Waters Corporation, Milford, MA, USA). Since the analysis focused on free amino acids, no hydrolysis was performed. Samples corresponding to different process time points were ultracentrifuged for 3 min at 12000 rpm. Briefly, a 70 μL volume of AccQFluor borate buffer was added to a 10 mL volume of sample each in a pyrolyzed derivatization tube. After vortexing for 10 s approximately, 20 μL of AccQFluor reagent were added and the mixture was vortexed for 10 s, and incubated for 1 min at room temperature. The excess reagent was then hydrolyzed to 6-Aminoquinolone (AMQ), *N*-hydroxysuccinimide (NHS) and carbon dioxide. None of these byproducts interfered with the analysis (data not shown). The solution was placed on a heating block at 55 °C for 10 min. The blank was derivatized by mixing and vortexing 80 μL of AccQFluor borate reagent and 20 μL AccQFluor reagent. A seven point standard calibration curve was constructed by successive dilution and analysis of a Waters Amino Acid Hydrolyzate Standard (a commercially available mixture that contains a 2.5 mM of each of the hydrolyzate amino acids and 1.25 mM of cysteine). The calibration standard was prepared by mixing 40 μL of the hydrolyzate amino acid standard with 960 μL of 18.2 MΩ water in a clean autosampler vial. The amino acid composition of the culture medium (CD OptiCHO™ medium) and the supplement (CHO CD EfficientFeed™ B) were also analyzed following this protocol.

### Quantification of amino acid derivatives

The amino acid derivatives obtained from the previously described procedure were chromatographically separated on a Waters 1525 chromatography system (Waters Corporation) coupled to a fluorescence detector (W2475; Waters 1525; Waters Corporation) using an excitation wavelength of 250 nm, an emission wavelength of 395 nm, a filter value of 0.5, and a gain value of 1.0. A 5 μL sample volume was injected for each analysis. The column used for the separation was a Waters AccQ.Tag Amino Acid Analysis Column, which is a high-efficiency Nova-Pak™ C_18_ (4 μm; 3.9 mm × 150 mm) certified for use with this method. A dual pump gradient system was implemented following a gradient of Eluent A (an acetate-phosphate buffer prepared by mixing 100 mL AccQTag Eluent A concentrate with 1 liter 18.2 Megohm water) and Eluent B (composed of 60 % acetonitrile and 40 % in 18.2 Megohm water) (see Table 1). Both mobile phases were filtered and degassed before the analysis. The column temperature was set at 37 °C (before starting the pump system), and was conditioned with an ACN solution (60:40 in water) for 5 min at a flow rate of 1 mL/min. Afterwards, the column was equilibrated for 9 min at a 1 mL/min flow with 100 % of Eluent A. The overall run time for the analysis using this methodology was 45 min (38 min run, 7 min delay).

**Table 1:** Gradient table for the dual pump HPLC system

Time	Flow rate (mL/min)	% A	% B
Initial	1.0	100	0
0.5	1.0	98	2
15.0	1.0	93	7
19.0	1.0	90	10
32.0	1.0	67	33
33.0	1.0	67	33
34.0	1.0	0	100
37.0	1.0	0	100
38.0	1.0	100	0

## Results and discussion

### General rationale of the experiment

Previous experimental work from our group resulted in the establishment of a feeding protocol to sustain growth and production of a recombinant CHO cell producer of a monoclonal antibody. Here, we estimated the amino acid concentration profiles of both naïve and recombinant cell lines in a batch process using a single initial addition of nutrients (at day 0) or the previously described supplementation protocol. We comparatively monitored the concentrations of essential and non-essential amino acids in culture experiments with naïve and recombinant CHO cells. This information provided insight into the role of amino acids in the processes of cell growth, cell maintenance, and monoclonal antibody production. Specifically, from the experiments with naïve cells, we determined the amino acid (essential and non-essential) consumption baseline. Then, from culture experiments with the recombinant cells, we inferred the additional demand of amino acids due to recombinant protein production. Since a relatively high-producing clone was used (final concentrations of 700 mg/L of mAb in 15 days of fed-batch culture), we observed significantly different amino acid demands during the first phase of the culture (dominated by growth) and a second stage (dominated by production).

### Growth curves for naïve and recombinant CHO cells

**Fig. 2:**
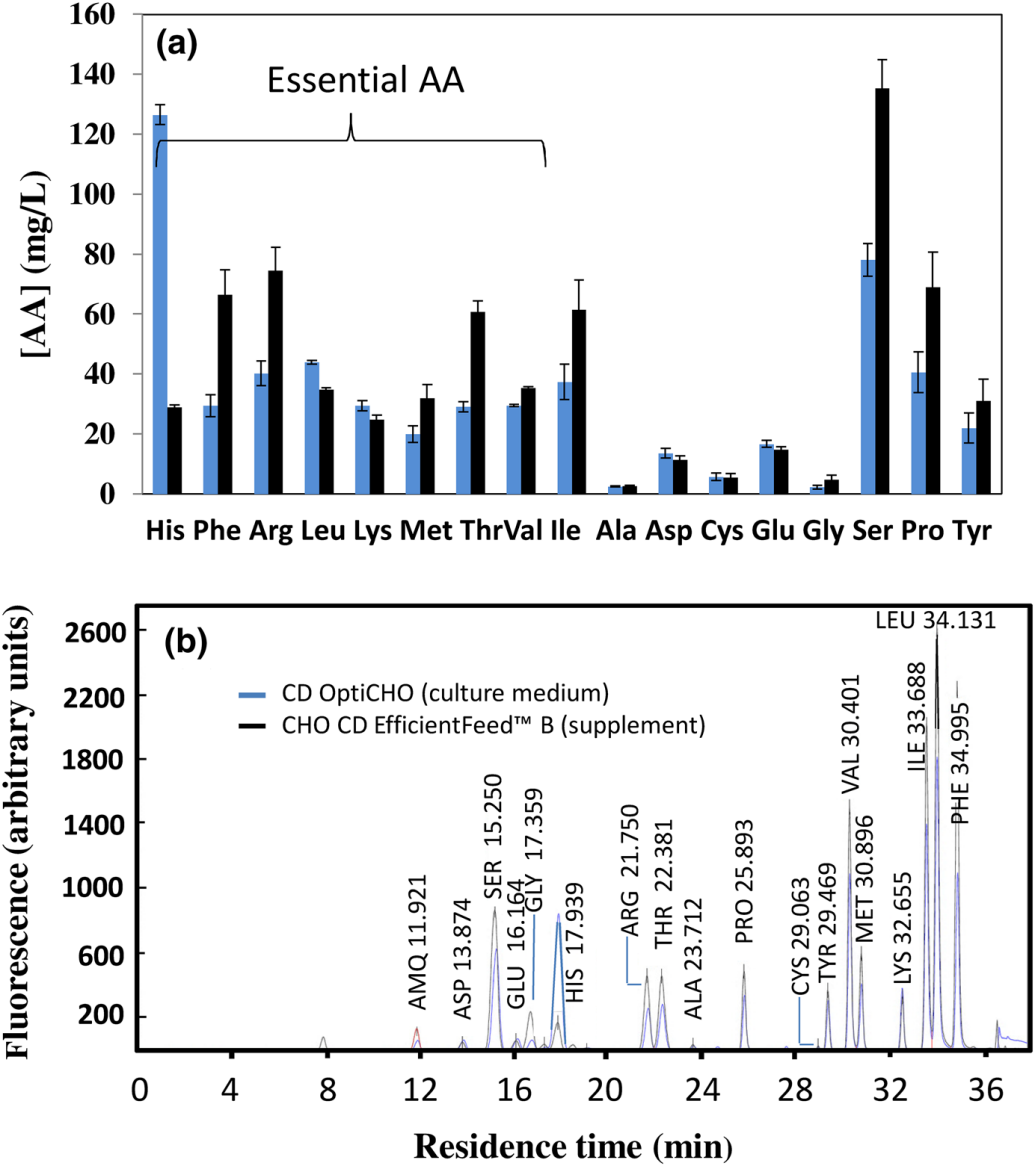
**a** Amino acid concentration in CD OptiCHO™ medium (*blue bar*), and CHO CD EfficientFeed™ B supplement (*black bar*), as determined using an AccQ-Tag pre-column derivatization technique followed by HPLC coupled to fluorescence detection. *Error bars* indicate variation from two repeats. **b** Representative chromatograms comparing the amino acid profile of CD OptiCHO™ medium (*blue line*), and CHO CD EfficientFeed™ B supplement (*black line*). The typical residence time for each amino acid peak is indicated. (Color figure online)

**Fig. 3:**
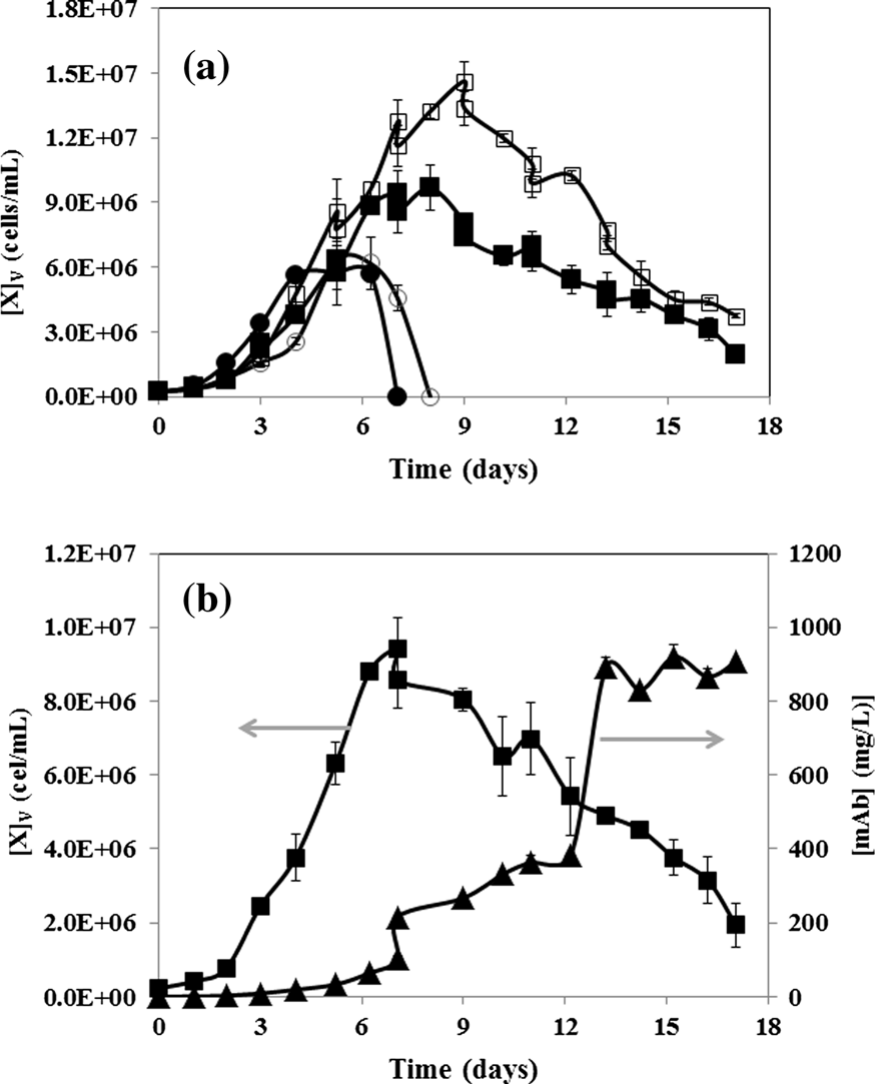
**a** Cell counts in CHO cell cultures of naïve cells cultured in batch mode without supplementation (*open circle*); naïve cells cultured in fed-batch mode with CHO CD EfficientFeed™ B supplementation (*open square*); mAb producers cultured in fed-batch mode without supplementation (*filled dot*) and; mAb producers cultured in fed-batch mode with CHO CD EfficientFeed™ B supplementation (*filled square*). **b** Cell count and mAb concentration profiles in fed-batch cultures of recombinant cells supplemented with CHO CD EfficientFeed™ B. *Error bars* indicate standard deviation from three repeats

Chinese hamster ovary cell culture media are primarily formulated to sustain adequate cell growth, but not necessarily recombinant protein production. For the second purpose, chemically defined supplements are used. Figure 2 presents the amino acid composition of the culture medium and the supplement used in our experiments, CD OptiCHO™ and CD EfficientFeed™ B respectively. As expected, commercial culture media and supplements are richer in essential amino acids. However, note that some non-essential amino acids (SER, PRO and TYR) are also present in a relatively high concentration. Figure 3 shows the cell count profiles over time for culture experiments in which naïve or recombinant cells were grown in medium un-supplemented or supplemented with CHO CD EfficientFeed™ B. In our batch experiments, naïve (Fig. 3a) and recombinant CHO cells survived for fewer than 8 days in un-supplemented medium. The maximum cell concentrations reached by naïve and recombinant cultures in un-supplemented media were 1.02 × 10^7^ and 7.68 × 10^6^ cells/mL respectively, which suggest that recombinant protein production demands significantly lowered the availability of amino-acids to sustain proliferation growth. Later in this communication, we will examine the specific differences in amino acid consumption among naïve and recombinant cells. Supplementation with CHO CD EfficientFeed™ B significantly enhanced the capacity of the culture to survive for longer times. Naïve and recombinant cell cultures survived for 7 and 17 days, respectively. As shown in Figs. 3a, b, the maximum cell density for naïve and recombinant cultures was also significantly different. Supplemented naïve cell cultures reached an average maximum cell concentration of 1.4 × 10^7^ cells/mL. Supplemented recombinant cultures reached a maximum cell density of 0.9 × 10^7^ cells/mL.

The cell count (three repeats) and mAb concentration profiles over time for the culture scenario in which recombinant cells were grown in supplemented medium is presented in Fig. 3b. This plot clearly distinguishes three regimes or process stages. During the first period, from inoculation to day 6, cell growth dominates the process. Cell counts increase approximately tenfold during the exponential phase, while product accumulation remains practically negligible. Therefore, at this stage, most of the amino acid demands can be attributed to cell proliferation. In the second stage, from day 6 to day 13, the product accumulates practically in a linear fashion. During this period, the cell concentration achieves a maximum (at day 7) and then decreases progressively. Therefore, during this stage, amino acid consumption is directed only to cell maintenance and product assembly. In the third stage, from day 13 to the end of the culture period, the product concentration reaches a plateau at its maximum concentration, although viable cell concentrations above 1 × 10^6^ cells/mL can still be observed. During this third stage, exhaustion of at least one amino acid can be inferred to limit the production of the monoclonal antibody.

More information can be extracted from the analysis of each amino acid concentration profile for each one of the four cases studied: (a) naïve cells growing in batch culture; (b) naïve cells cultured in fed-batch mode with supplemented medium; (c) recombinant cells growing in batch culture; and (d) recombinant cells cultured in fed-batch mode with supplemented medium.

### Consumption of essential amino acids

We analyzed the concentration profiles for each amino acid considered essential for CHO cells; that is, amino acids that must be supplied in the culture media since CHO cells are not able to synthesize them de novo. As expected, each essential amino acid was consumed to a different extent by naïve and recombinant cells. Some specific observations follow: In Table 2, we show the extent of conversion (expressed as a fraction with respect to initial concentration) of each essential amino acid in naïve cells and recombinant producers. In all cases, consumption has been calculated based on final concentrations of amino acids in 7 days batch experiments. Conversions have been ordered from the highest to the lowest. HIS, PHE, and ARG show the highest degree of conversion in naïve cells (see also González et al. 2011). In particular, more than 95 % of the supplied HIS is consumed in our batch experiments. The rest of the essential amino acids, LEU, LYS, MET, THR, VAL, and ILE showed conversions below 0.80, which suggest that these are dosed in proper amounts in the culture medium used. Evidently, amino acid consumption is higher in recombinant cells.

**Table 2:** Conversion of essential amino acids in naïve and recombinant CHO cells during batch experiments

AA	Mass fraction converted		Δ in conversion	% in mAb
	Naïve cells	Recombinant cells		
HIS	0.96 ± 0.0009	0.97 ± 0.0007	0.01	3.33
PHE	0.87 ± 0.0140	0.92 ± 0.0092	0.04	5.22
ARG	0.87 ± 0.0120	0.91 ± 0.0086	0.04	4.4
*LEU*	0.77 ± 0.0034	0.84 ± 0.0024	*0.07*	8.611
*LYS*	0.75 ± 0.0137	0.84 ± 0.0087	*0.09*	8.131
**MET**	0.75 ± 0.0300	0.83 ± 0.0200	0.08	1.508
*THR*	0.74 ± 0.0144	0.82 ± 0.0010	*0.08*	8.131
*VAL*	0.76 ± 0.0027	0.82 ± 0.0020	*0.06*	9.175
**ILE**	0.70 ± 0.0412	0.80 ± 0.0280	0.10	3.472

In general, the amino acids with the highest differential conversion (italics) are those which constitute 6 % or more of the molar mass of the recombinant protein. The amino acids with the highest conversion had values higher than 0.80. The exceptions are ILE and MET (indicated in bold)—amino acids with high differential conversion and relatively low presence in the molecular structure of the mAb

As expected, the increment in conversion due to recombinant protein production—that is, the ‘Δ in conversion’ in Table 2—differs for each amino acid. For example, although HIS is the amino acid that undergoes the highest conversion in both naïve and recombinant cells, its increase in conversion due to recombinant protein synthesis is the lowest among all essential amino acids (less than 0.01). This is because HIS represents only the 3.3 % of the molecular mass of the anti-TNFα mAb.

The differences in conversions (between recombinant and naïve cell cultures) of the essential amino acids that are more frequent in the molecular structure of the mAb are expected to be higher. This is precisely the case for LYS, THR, LEU, and VAL, whose differences in conversions were 0.09, 0.08, 0.07, and 0.06 respectively. Consistently, these are the four essential amino acids that are most frequently present in the structure of the recombinant protein, constituting 8.13, 8.13, 8.61, and 9.17 % of the total mass of this mAb, respectively. Interestingly, our results suggest that ILE and MET are demanded by producer cells to sustain the synthesis of the mAb. ILE and MET also exhibited high differences in conversions between recombinant and naïve cultures, at 0.10 and 0.08, respectively, although they represent only 3.47 and 1.50 % of total mass of the mAb (see Table 2).

**Fig. 4:**
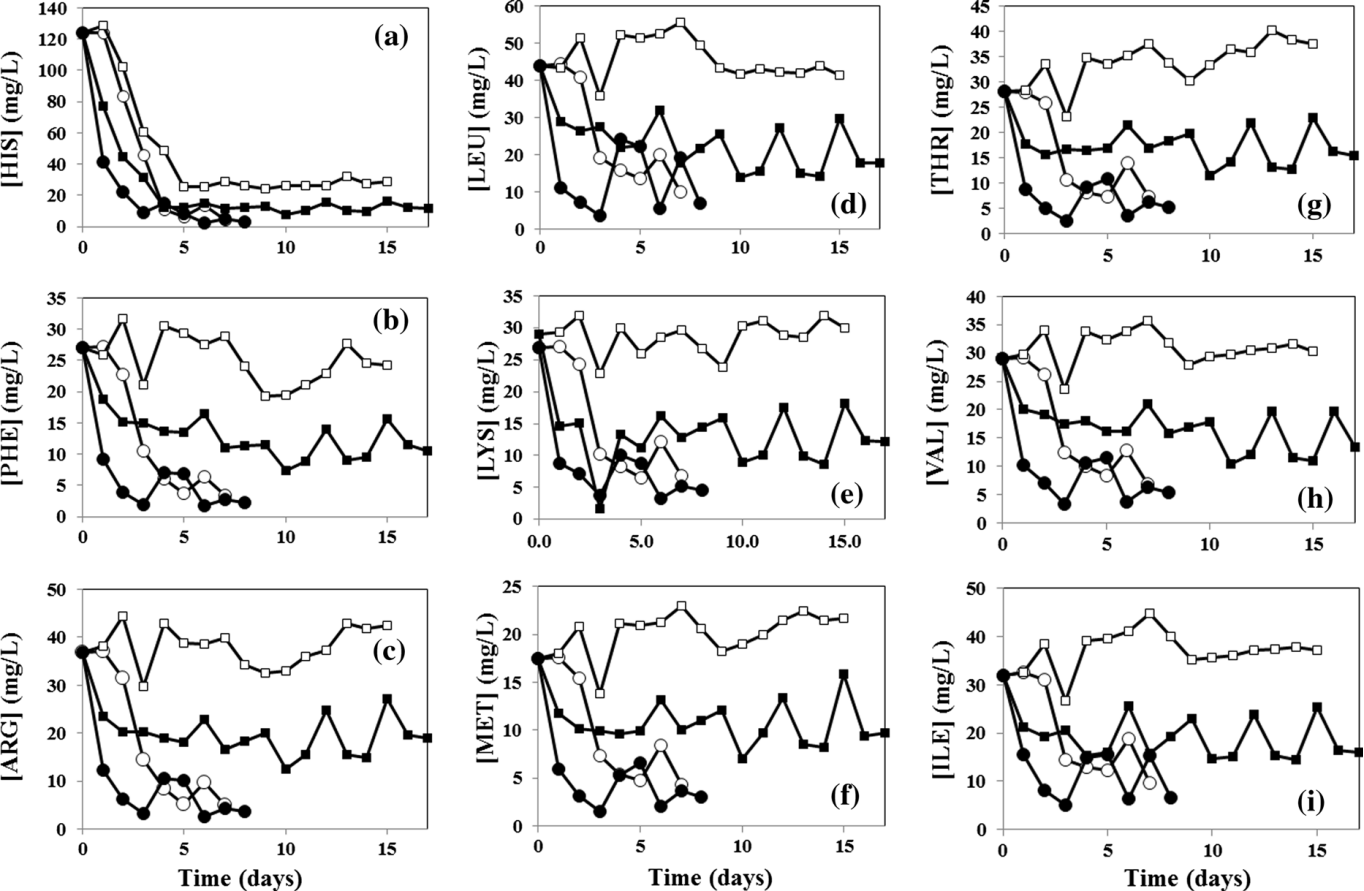
Concentration profiles of each essential amino acid during unsupplemented batch cultivation of naïve CHO cells (*open circle*), unsupplemented batch cultivation of recombinant CHO cells (*filled circle*); supplemented fed-batch cultivation of naïve CHO cells (*open square*); and supplemented fed-batch cultivation of recombinant CHO cells (*filled square*)

Figure 4 shows the concentration evolution of essential amino acids for naïve and recombinant cells, growing in un-supplemented or supplemented CD OptiCHO™ culture medium. In the case of unsupplemented growth of naïve cells (empty circles), the decrease in concentration observed in the first 7 days is due only to cell growth. For the case of unsupplemented growth of recombinant cell culture (black circles), the product begins to accumulate only after the fifth day. Therefore, the contribution of amino acid demands for mAb production is only marginal up to day 5. However, as previously shown (Table 2), even during this first stage of the process, a higher conversion of all amino acids was observed in the case of recombinant cell culture experiments. In this particular scenario (culture of recombinant CHO cells with no supplementation), a significant experimental error exists in the samples corresponding to days 4 and 5. However, for data completeness, we decided not to discard these values. This error does not interfere with the calculation of final conversions, which considers the final point of the experiments (corresponding to days 7 and 8).

In the case of supplemented cell culture of naïve cells, the amino acid cell consumption due to growth and cell maintenance is alleviated by the supply of fresh CHO CD EffcientFeed™ B every second day, which contributes amino acids among other components. In these experiments, the maximum cell concentration exceeds a value of 1.4 × 10^7^ cells/mL at day 9, a value much higher than the one observed in experiments with recombinant cells.

Much lower maximum cell concentrations were recorded for the case of supplemented culture experiments with recombinant cells (an average of 0.9 × 10^7^ cells/mL), since the demand for amino acids for product synthesis competes with that for cell growth. Our results suggest that the implemented protocol of addition is sufficient to conveniently address the needs of cell growth, since the experiments with naïve cells showed a final concentration of all essential amino acids maintained in the range of 20–45 mg/L. However, in the culture experiments with mAb producer cells, the concentrations of most essential amino acids fall in a lower range of values, from 10 to 15 mg/L, with HIS and PHE as the two amino acids with the lowest residual concentrations. Interestingly, HIS and PHE together constitute only 5.55 % of the total mass of the anti-TNFα mAb. Using the supplementation strategy described, no apparent shortage of LEU, LYS, THR, or VAL—amino acids that account for the 33 % of the total mAb mass—was observed during the 15 day culture period.

### Consumption of non-essential amino acids

**Fig. 5:**
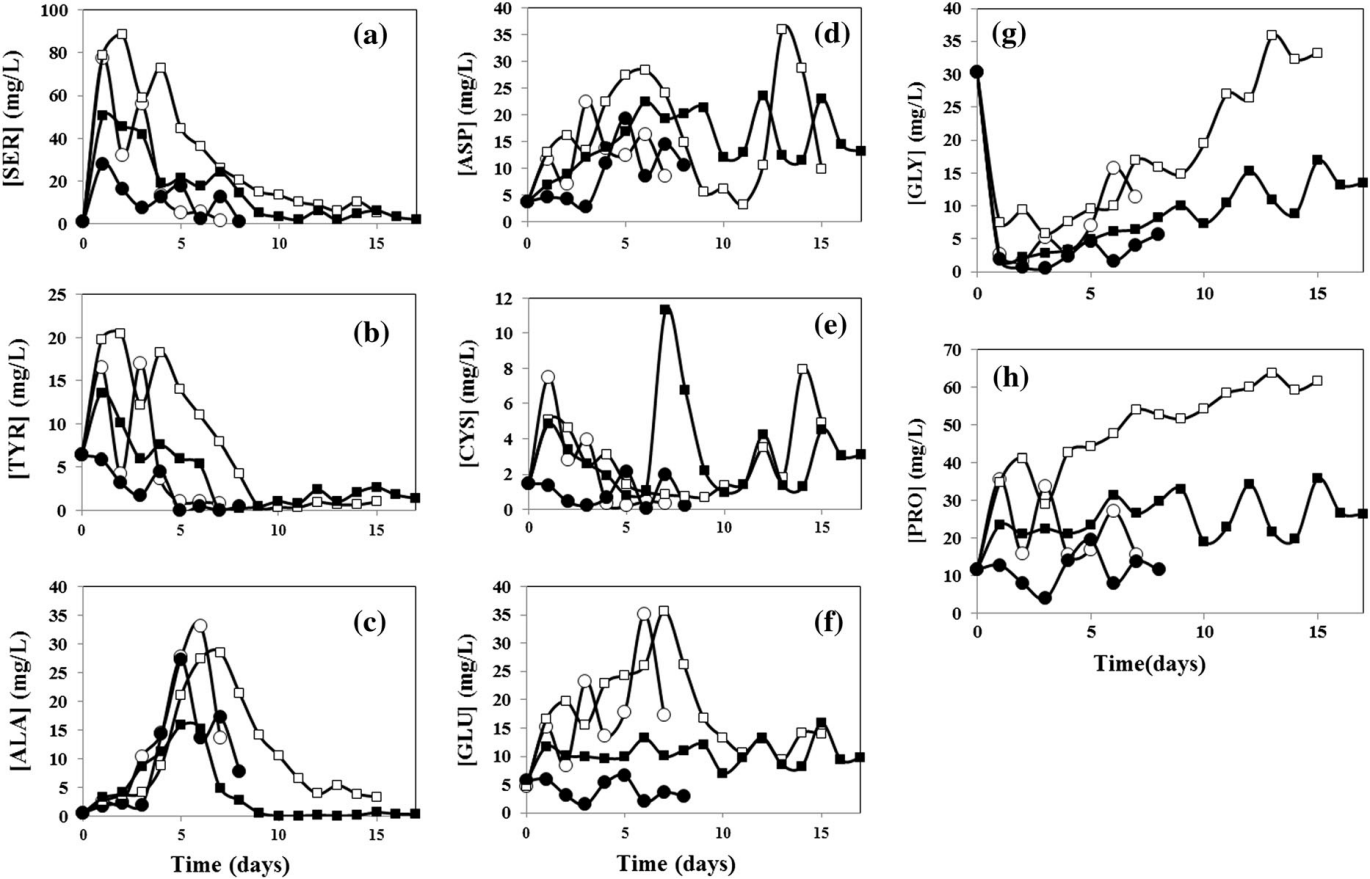
Concentration profiles of each non-essential amino acid during unsupplemented batch cultivation of naïve CHO cells (*open circle*); unsupplemented batch cultivation of recombinant CHO cells (*filled circle*); supplemented fed-batch cultivation of naïve CHO cells (*open square*); and supplemented fed-batch cultivation of recombinant CHO cells (*filled square*)

The cell is capable of synthesizing non-essential amino acids to fulfill, partially or completely, its growth needs. In the case of non-recombinant mammalian cells culture, amino acid production may not be sufficient to balance amino acid consumption and more supplementation could be needed. The changes in concentration of nonessential amino acids, in the particular case of the cell line we have used, revealed three different scenarios. The amino acids ALA, TYR, and SER, were completely depleted from the culture media, which indicates that their supplementation was not sufficient (Fig. 5a–c). A second group of amino acids (ASP, CYS, GLU) reached a steady state concentration, which suggest that a balance was reached between consumption and production plus supplementation (Fig. 5d–f). Two amino acids, specifically GLY and PRO, accumulated in the culture medium, indicating that their supplementation was excessive (Fig. 5g–h).

The non-essential amino acids ALA, TYR, and SER, which were completely exhausted from the culture medium, are of particular interest. SER and TYR are important constituents of the mAb, representing the 12.7 and 6.40 % of the mAb mass composition, respectively. Consequently, they are in high demand in mAb producers. In our experiments with supplementation protocols, SER is not completely exhausted from the medium by naïve cell cultures (see Fig. 5a) but is almost entirely consumed by recombinant cells after day 10. This suggests that SER supplementation is sufficient to sustain growth, but not mAb production. TYR is fully depleted after day 9 in both naïve and recombinant cells (Fig. 5b). This indicates that TYR supplementation is insufficient to even sustain growth of this particular cell line. In contrast, ALA shows no full depletion in batch or supplemented experiments with naïve cells. In the supplementation protocols, even after 15 days of culture of mAb producers, some residual ALA was measured. ALA constitutes only 3.84 % of the mass of the mAb. However, in the supplemented recombinant cell culture, ALA concentration rapidly fell to practically zero concentration after day 9. This result suggests that ALA is important in the metabolic processes related to recombinant protein synthesis, although it is not a main constituent of this particular mAb.

Of the group of amino acids that apparently reach a steady state concentration, CYS deserves a special mention. Even though CYS is a non-essential amino acid in terms of human nutrition, it has been considered essential for mammalian cells in culture (Hu 2012). In our supplemented experiments, CYS concentration was maintained at low levels (below 10 mg/L) in both naïve and recombinant cells. Although CYS does not appear to be limiting growth or production, further supplementation with CYS could be advisable.

PRO and GLY accumulated in the culture medium of fed-batch experiments with naïve cells. However, in experiments with recombinant cells, the concentration of PRO and GLY reached steady state values of 25 and 13 mg/L, respectively. PRO is one of the most abundant non-essential amino acids in the CD OptiCHO™ medium formulation. The accumulation of GLY, however, is mostly due to cell production, since GLY is not present in high concentrations in the culture medium.

## Conclusions

In this work, we quantified and discussed the amino acid demands due to cell growth and recombinant protein production during long term fed-batch cultivation protocols for a specific high-producing mAb CHO cell line.

This study shows the results of amino acid composition analysis from cell culture media obtained throughout the duration of the culture by way of a straightforward and relatively simple HPLC-FLD methodology via the Waters ™ AccQTag pre-column derivatization technique. The determination of basal amino acid content from both the commercial culture medium and supplement allowed the comparison of these two media and the different stages of our cell culture experiments. Consequently, we were able to identify which amino acids are demanded at every stage of growth, maintenance, and production in naïve and recombinant cell lines. This knowledge will eventually allow cell culture experiments to be optimized with said amino acids in order to enhance growth and mAb production.

In the experiments reported in this work, the time concentration profiles of essential and non-essential amino acids were monitored in cultures of naïve and recombinant CHO cell cultures producers of a monoclonal antibody.

Based on our experiments, we cannot establish clear statistically significant differences in consumption rates. However, we believe that our results are useful for analyzing general trends and for conducting a relative comparison between treatments. We observe that certain behaviors are consistent among the data set. In particular, the naïve cell cultures showed the highest degree of conversion for HIS, PHE, and ARG among essential amino acids. In particular, more than 95 % of the supplied HIS is consumed in our batch experiments. As expected, amino acid consumptions are higher in recombinant cells; a higher demand was seen for LYS, THR, LEU, and VAL, which are the essential amino acids that are the main constituents of the mAb. Interestingly, ILE and MET are also in high demand by producer cells although they represent only 3.47 and 1.50 % of total mass of the mAb. This suggests that ILE and MET are needed to sustain the biochemical machinery of synthesis of recombinant proteins in CHO cells. Among the non-essential amino acids, ALA, TYR, and SER were completely depleted from the culture medium, which indicates that their supplementation was insufficient. A second group of amino acids, namely ASP, CYS, GLU, reached a steady state concentration, which suggests that a balance between consumption and production plus supplementation was reached. Two other amino acids, GLY and PRO, accumulated in the culture medium, indicating that their supplementation was excessive. Of the non-essential amino acids (ALA, TYR, and SER) that were completely exhausted from the culture medium, SER and TYR are important constituents of the structure of the recombinant protein, representing the 12.7 and 6.40 % of the mAb mass composition, respectively. The rapid depletion of ALA, an amino acid that constitutes only 3.84 % of the mass of the mAb, suggests that ALA is important in the metabolic processes related to recombinant protein synthesis, although it is not one of the main constituent of this particular mAb.

This study shows that the evaluation of the demands for specific amino acids provides valuable information for the rational design (or evaluation) of CHO cell culture medium and optimization of feeding/supplementation strategies.
